# Last rolls of the yoyo: Assessing the human canonical protein count

**DOI:** 10.12688/f1000research.11119.1

**Published:** 2017-04-07

**Authors:** Christopher Southan

**Affiliations:** 1 IUPHAR/BPS Guide to Pharmacology, Centre for Integrative Physiology, University of Edinburgh, Edinburgh, EH8 9XD, UK

**Keywords:** proteins, genes, human genome, proteomics, mass spectrometry

## Abstract

In 2004, when the protein estimate from the finished human genome was only 24,000, the surprise was compounded as reviewed estimates fell to 19,000 by 2014. However, variability in the total canonical protein counts (i.e. excluding alternative splice forms) of open reading frames (ORFs) in different annotation portals persists. This work assesses these differences and possible causes. A 16-year analysis of Ensembl and UniProtKB/Swiss-Prot shows convergence to a protein number of ~20,000. The former had shown some yo-yoing, but both have now plateaued. Nine major annotation portals, reviewed at the beginning of 2017, gave a spread of counts from 21,819 down to 18,891. The 4-way cross-reference concordance (within UniProt) between Ensembl, Swiss-Prot, Entrez Gene and the Human Gene Nomenclature Committee (HGNC) drops to 18,690, indicating methodological differences in protein definitions and experimental existence support between sources. The Swiss-Prot and neXtProt evidence criteria include mass spectrometry peptide verification and also cross-references for antibody detection from the Human Protein Atlas. Notwithstanding, hundreds of Swiss-Prot entries are classified as non-coding biotypes by HGNC. The only inference that protein numbers might still rise comes from numerous reports of small ORF (smORF) discovery. However, while there have been recent cases of protein verifications from previous miss-annotation of non-coding RNA, very few have passed the Swiss-Prot curation and genome annotation thresholds. The post-genomic era has seen both advances in data generation and improvements in the human reference assembly. Notwithstanding, current numbers, while persistently discordant, show that the earlier yo-yoing has largely ceased. Given the importance to biology and biomedicine of defining the canonical human proteome, the task will need more collaborative inter-source curation combined with broader and deeper experimental confirmation *in vivo* and *in vitro* of proteins predicted *in silico*. The eventual closure could be well be below ~19,000.

## Introduction

While hypothesis-neutral scientific endeavours are sometimes referred to in derogatory terms as “stamp collecting”, the collation of molecular part lists (e.g. genes, transcripts, proteins and metabolites) remains a crucially important exercise, not only for many aspects of basic biology, but also for application to the biomedical sciences and drug discovery. Paradoxically, however, despite technical advances in analytical experimentation that should be making them easier to verify and quantify, definitive (or “closed”) counts of even just these four entities for key species remain largely refractive. This is particularly so for proteins, as the most demonstrably biologically functional of these entity sets, even though they were the first to emerge historically by many decades ^1^. In 2001, an analysis of the first public version of the draft human genome included an estimate of ∼24,500 protein-coding genes ^2^. The general opinion at that time was that this was lower than expected and would thus probably rise above 30,000. Notwithstanding, when the more complete first reference assembly (92% euchromatic coverage at 99.99% accuracy) was released in May 2004, the estimate was revised slightly downwards to ∼24,000 ^3^. In the same year a detailed review appeared supporting a lower bound of ∼25,000 ^4^. This latter publication alluded to a “yoyo” effect that persisted in subsequent reviews by falling to ∼20,500 in 2007 ^5^, rising to 22,333 in 2010 ^6^, but then dropping to ∼19,000 by 2014 ^7^. Those accepting the latter estimate may have felt a touch of chagrin as the count thereby fell to ∼ 1000 below the model worm *Caenorhabditis elegans*. While we humans were still, reassuringly perhaps, ∼ 7000 proteins ahead of the model fly *Drosophila melanogaster*, we are still ∼20,000 behind the lowly *Paramecium* (see Table 1).

**Table 1.** Human protein coding gene counts from nine different portals, collected at the beginning of 2017. Ensembl numbers for the yeast, worm, fly and a protozoan are included for comparison (abbreviations are defined in the text).

Source	Version/date	Total	URL
GeneCards	v4.3.4, Jan 2017	21,819	http://www.genecards.org/
GeneID	Feb 2017	20,671	https://www.ncbi.nlm.nih.gov/gene/statistics/
Swiss-Prot	Release 2017_01	20,171	http://www.uniprot.org/
neXtprot	Jan 2017	20,159	https://www.nextprot.org/about/statistics
GENECODE	v25, March 2016	19,950	http://www.gencodegenes.org/stats/current.html
Ensembl	87.38	19,915	http://www.ensembl.org/Homo_sapiens/Info/Annotation
Vega/Havana	Feb 2017	19,768	http://vega.sanger.ac.uk/Homo_sapiens/Info/Annotation
HGNC	Feb 2017	19,033	http://www.genenames.org/cgi-bin/statistics
CCDS	20, Aug 2016	18,891	https://www.ncbi.nlm.nih.gov/projects/CCDS/CcdsBrowse.cgi
*S. cerevisiae*	Dec 2011	6,692	http://www.ensembl.org/Saccharomyces_cerevisiae/Info/Annotation
*C. elegans*	WS250, 2012	20,362	http://www.ensembl.org/Caenorhabditis_elegans/Info/Annotation
*D. melanogaster*	Release 6, 2014	13,918	http://www.ensembl.org/Drosophila_melanogaster/Info/Annotation
*P. tetraurelia*	v87.1, 2006	39,642	http://protists.ensembl.org/Paramecium_tetraurelia/Info/Annotation/#assembly

This article will compare and discuss the current numbers (as of 1Q 2017) from major sources. The evidence types and theory behind protein counting have been described in many publications and documentation from the individual database portals, but the reviews referenced above provide complementary background. It needs to be stated that numbers used herein refer to what can be termed the “canonical” human proteome. This has its origins in the Swiss-Prot approach to protein annotation whereby protein sequence differences arising from the same genomic locus either by alternative splicing or alternative initiations (or permutations of both) and/or genetic variants, are all cross referenced to a single, maximal length, protein entry ^8^. Importantly, while this was originally introduced as the curatorial strategy of choosing the longest mRNA for an entry, it actually turns out to have post-genomic data support, not only in the form that coding-loci express a single main protein (i.e. that most predicted alternative transcripts may not be translated), but also that in most cases this is the max-exon form (i.e. the curatorial choice actually seems to be the biological “default”) ^9^.

## Historical growth

**Figure 1.**
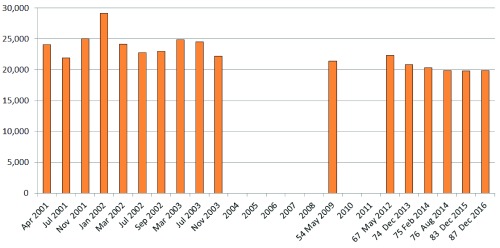
Protein counts from the Ensembl pipeline database releases over the first three years and last seven years. The latter are only those from the current archive that have protein rebuilds rather than maintenance/patch releases with nearly identical numbers.

The set of open reading frames (ORFs) constituting the canonical human proteome can be historically followed in Ensembl and Swiss-Prot (as the manually reviewed and expert annotated sub-set of UniProtKB). Both of these are very different pipelines, but are partially coupled in the sense that the latter is one of the inputs to the automated ORF-building algorithms of the former. We can assess the progress of Ensembl first, since it has been compiling an approximation to the human proteome based on genomic predictions since 2001 ^10^. A 2004 review assessed historical figures from the first three years, over which the total shifted only marginally from 24,037 to 24,046 ^4^. While a maximum of 29,181 was reached in January 2002, this was an artefact associated with clone orientation changes caused by a switch in the assembly source, and this number had dropped back to 24,179 by the next release. Despite some year gaps (not covered by the current archived data sets) the older figures can be plotted with the most recent ones to give a 15 year-span ( Figure 1).

It is important to note that, for technical reasons, the longitudinal Ensembl protein numbers are not strictly comparable, since the pipeline model, its parameterisations and data feeds, have, as one might expect, evolved considerably over the years (e.g. the assembly source change mentioned above). This has included incremental improvements of various kinds (e.g. in the quality of the reference genome), but some changes have altered the exact definitions of the headline protein numbers. For example, the pseudogene figures given in the early 2001–3 releases needed to be subtracted from the totals. Those earlier numbers also specified a proportion of novel genes (defined as not having an exact match to RefSeq or UniProt entries at build time), but these tailed off from a maximum of 12,398 in November 2001 to only 46 by 2009 (release 54).

The most recent releases have other changes that complicate protein counts. One of these is the inclusion of “alternative sequence”, referring to genomic sections that differ from the primary contiguous assembly. The current release of Ensembl (87.38) specifies 2,541 proteins in this category, but it is not clear which of these are just variants of those derived from the primary assembly. Another, somewhat enigmatic aspect, is the appearance in the protein count of so called “read-through” genes. These are defined as transcripts connecting two independent loci on the same strand. These debuted at 463 in release 74, via manual annotation, climbing slowly to the current total of 526. While they are also included in the NCBI genome annotation, these have not been included in the Figure 1 counts because, if they exist at all as translated chimeric proteins, they are non-canonical by definition. Despite these shifts in exactly what the protein numbers represent, we can draw three principle conclusions from Figure 1. These are: a) yo-yoing has at least subsided, if not ceased; b) the number has plateaued at just below 20,000; and c) the pipeline has ceased to spawn significant numbers of novel proteins (i.e. they are now predominantly “seen before”).

**Figure 2.**
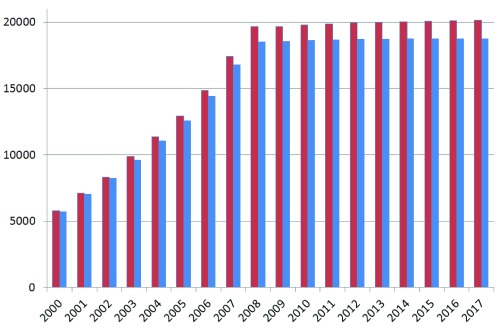
Protein counts from human UniProtKB/Swiss-Prot by create date (red). The blue columns include the additional selection for existence evidence at the protein or transcript levels (note the date is just for the entry into Swiss-Prot, not the first appearance of the sequence in TrEMBL that can be many years earlier).

One of the core operations for Ensembl is resolving transcripts and their mRNA coding sections (CDSs) against ORFs predicted *ab initio*. Swiss-Prot, on the other hand, has historically been doing this for mRNA-to-protein independently of genomic coordinates (although it increasingly now maps the two together where possible). Over the years, the criteria and manual triage for defining canonical ORFs have been consistently applied in Swiss-Prot. This means the growth rate can be straightforwardly recorded by slicing Swiss-Prot human proteins by “create date” ( Figure 2). The pattern is interpretable as a concerted effort towards provisional closure of the proteome at 19,658 by 2008. Subsequent increases were essentially incremental, climbing slowly to 20,168 by 2017.

While issues around evidence types will be addressed later, a simple filter can be applied to count just those proteins with either transcript and/or other forms of experimental support for their existence. The result, in the Figure 2 plot, shows this difference to be fairly constant (i.e. that in the order of ∼1,400 sequences remaining experimentally unsupported). There are three other salient features. The first is that the total has only increased by a modest 516 since 2009, whereas Ensembl shrunk by 1,455 over the same period. They have thus both converged towards ∼20,000 (it is not clear if the two sets are congruent for the same ORFs, but this question will be addressed later). However, there were already indications of approximate concordance as early as 2001, where adding the Ensembl novels to the Swiss-Prot knowns reached 18,191. The inference is that the number of novel proteins confirmed since 2001 is less than 2000. Note also that many are TrEMBL-to-Swiss-Prot promotions (i.e. with data already surfaced) rather than *de-novo* deposited protein sequences. By comparing 2009 with the subsequent seven years we can also infer that Swiss-Prot has not purged significant numbers of accessions (i.e. they have revised sequences but generally not removed them).

## Current counts

We can move on from tracking historical numbers to taking a contemporary snapshot of major sources (including the two already described) that are well established and regularly declare revised protein counts ( Table 1). There are many aspects that could be expanded on from this set, but the feature that immediately stands out is the difference of nearly 3000 between highest and lowest (i.e. 13%). The highest figure comes from what can be considered a meta-source, GeneCards, that merges different pipeline outputs, so this could be expected to be an upper bound ^11^. The protein-coding set from the NCBI genome annotation pipeline ranks second but there are some caveats regarding comparability with the other sources ^12^. One of these is the inclusion of 1235 “LOC” entries with low homology support. Although 107 of these do have Ensembl gene IDs, none have been assigned Human Gene Nomenclature Committee (HGNC) symbols. Removing LOCs from the NCBI protein set would drop them down to seventh at 19,436.

The next two sources are related in that neXtprot takes the human Swiss-Prot set as a starting point for evidence expansion and interrogation enhancements. This is why these have (almost) the same count (the residual differences being due to synchronisation timings) ^13^. The next three sources are also coupled in the sense that not only are GENECODE and Vega marked-up in Ensembl, but there are plans to merge the three. However, they do show a small difference of 182, with the lowest being the Vega pipeline (as Havanna manual curation). But even from Vega, there is a substantial drop of 735 to the stringently reviewed approved protein-coding gene-based assignments from the HGNC. The lowest number in Table 1, coming in at just below 19,000, comes from the Consensus Coding Sequence (CCDS) project. These correspond to a core set of proteins annotated as having full length transcripts that exactly match reference genome coordinates.

Some sources have invested effort into mapping between each other’s identifiers. This can establish if the protein sequence in pipeline output A is the same as pipeline B. However, the fidelity of such a mapping (and consequent cross-reference reciprocity) depends on differences in methods and stringencies. For example, for all intents and purposes the beta-secretase 1 entry (BACE1) is the same across all 9 pipelines. However, a different population variant was chosen on each side of the Atlantic. Therefore, the RefSeq and Gene ID sequence NP_036236 differs by one residue (481 Cys → Arg) from the Swiss-Prot and Ensembl sequence as P56817. Note also that HNGC does not instantiate sequence entries in the way that the other pipelines do, but collates cross-references, so in this case HGNC 933/BACE1 points to both sequences. The process of cross-referencing between multiple annotation sources allows the generation of both intersects and differences. Crucially, in terms of protein counting, this gives us the possibility to discern where they are concordant or discordant and (on a good day) we may be able to identify causes for the latter.

## Cross-reference counting

**Figure 3.**
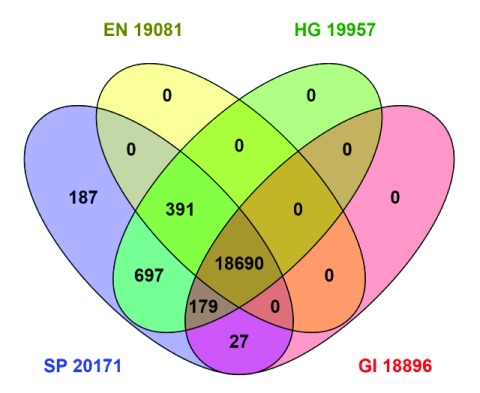
Intersects between identifier cross-references recorded from the UniProt interface. The results are generated via cross-reference totals according to UniProt, not from the sources *in situ*. EN, Ensembl; SP, Swiss-Prot; HG, Human Gene Nomenclature Committee; GI, NCBI Entrez Gene.

All nine sources in Table 1 provide some extent of cross-referencing between what should be the same protein in different sources (also referred to as cross-mapping). However, the choice was made here to exemplify just four identifiers, Swiss-Prot accession numbers, HGNC IDs (directly, or via the current gene symbols) Ensembl gene IDs and NCBI Entrez Gene IDs. These were chosen for their global prominence but also methodological complementarity. This derives from the fact that that the first two are essentially automated pipelines (but different), while the second two are primarily manual expert annotation operations (but also different). Each of the four offers their own internal ways of querying cross-references, including BioMart installations ^14^ or downloadable mapping tables for this to be done extrinsically. However, because it has the largest number of selectable cross-references, as well as extended options for live-linked result displays and filtered downloading, the UniProt interface was used here. Intersects for the four sources can be seen in Figure 3.

Figure 3 can be explained as follows: The queries executed gave the totals indicated in the segments. Note that some segments are empty, because, by definition, the identifier mapping has been done “inside” Swiss-Prot (even if in some cases the external sources collaborated in generating the mappings). By comparing with Table 1, we can thus see that 2,923 NCBI proteins did not map at all (which includes most of the LOCs). Similarly, 834 Ensemble protein gene IDs also did not map. For HGNC, on the other hand, we see the cross-reference result is actually 905 higher than the distinct identifier count at source. One explanation could be a proportion of a one-to-many relationship (e.g. Swiss-Prots with more than one HGNC). Some were identified, such as haemoglobin subunit alpha ( P69905) that maps to HGNC HBA1 and HBA2.

A notable result from Figure 3 is that a 1:1:1:1 mapping (i.e. four-way concordance) is achieved for only 18,690 proteins, lower than any of the totals from Table 1. Detailed analysis of all the segments cannot be presented here but some trends can be noted. Starting with the 187 in the “SP” segment (i.e. Swiss-Prot only, absent from the other three), the majority of the protein names are given as “putative” or “uncharacterised”. The 391 common elements in "SP", "EN" and "HG" (i.e. missing in NCBI Gene) are clearly dominated by variable domains of immunoglobulin light chains and HLA class I histocompatibility antigen alpha chains, the polymorphic nature of which necessitates a level of manual annotation that may not have been compatible with the NCBI pipeline automation. The 179 common elements in "SP", "HG" and "GI” (i.e. missing in Ensembl) are enriched for “Uncharacterized protein” from the so called Chromosome ORF predictions. The large set of 697 common elements in "SP" and "HG" (i.e. missing in NCBI Gene and Ensembl) are heterogeneous but show enrichment for translated endogenous retrovirus transcripts, putative uncharacterized proteins encoded by LINC loci and include 41 odour receptors. Notably, in these three sets, the HGNC cross-references classify them as not being within their own protein-coding set of 19,033, but rather as endogenous retrovirus, long non-coding RNAs and pseudogenes, respectively. This particular discordance (i.e. in UniProt but not a protein according to the HGNC) explains the 1: many cross-references mentioned at the start of this section. A duplicate check on the 960 indicated only 152 could be ascribed to Swiss-Prots with multiple HGNCs. It can also be seen in Figure 3 that two of the Swiss-Prot intersects are empty. The explanation is that Ensemble and NCBI Gene have consolidated mapping reciprocity for proteins in Swiss-Prot (but, as mentioned above, many proteins from these two sources are still nominally “outside” Swiss-Prot).

As one of its powerful utilities, we can interrogate ∼ 90 cross references in UniProt. While not all of these are human-relevant we can chose those to compare with Table 1. This has already been done for the four above but can be extended. For example, we can determine counts of 18,384 from CCDS and 19,940 for GeneCards. Note both of these are below the *in situ* counts by 510 and 1,871 respectively (GENCODE and Vega do not currently have cross-references inside Swiss-Prot). In some cases it may be possible to investigate counts reciprocally. For example, from the HGNC protein-coding download table we can establish that the 19,035 rows in the UniProt mapping column contained 18,997 Swiss-Prot IDs. The same table includes 19,035 Vega Gene ID mappings that also collapse to 18,973 distinct entries. This confirms what was implicated already above, as a small proportion of multiple Swiss-Prots < > HGNCs is also occurring for HGNC < > Vega. Cross-mapping counts can similarly be explored via other sources for comparison, depending on what query and/or download options are available. However, accumulating such results can quickly generate large Venn-type sets that generally end up being more confusing than illuminating.

Following on from above, since they are derived from structure data sources, cross-references give precise protein counts; but they also have associated equivocality (even though they will be used further in this report). For this reason, it is important to understand (e.g. via source documentation) technical differences in exactly how the mappings are determined. A second problem is they may be circular (i.e. source B may collegially accept A< > B mappings from source A without independently verifying the reciprocity of B >A). The third problem is synchronisation, where release dates are at different intervals (and may not always include mapping refreshes). The forth problem is the “churn” rate (appearance and/or disappearance of protein records) in genome resources. This is much lower that is was some years ago, but can still be an issue.

## Existence evidence

**Figure 4.**
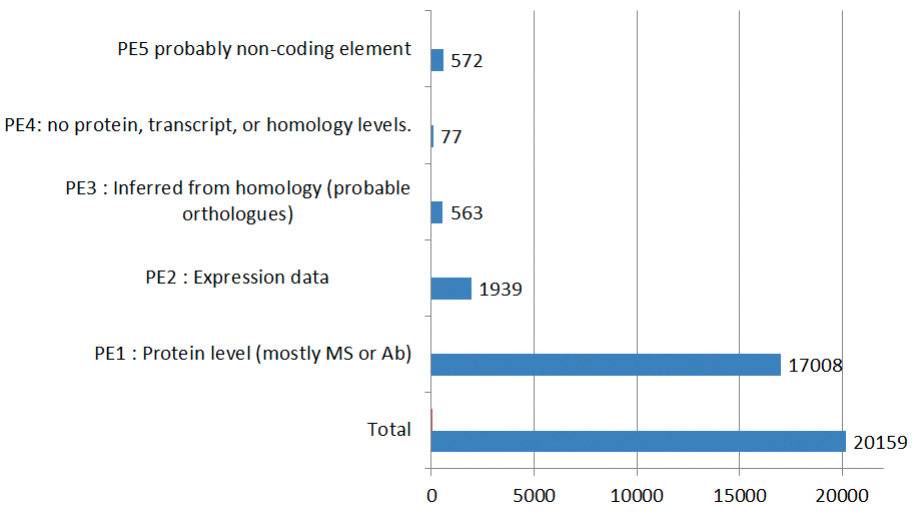
Protein existence codes and their occupancy statistics from neXtprot.

In the context of advancing towards proteomic “closure”, the imperative to verify the existence of an *in silico* database ORF as an *in vivo* protein translation product is obvious. By definition, the prerequisite mRNA transcription also needs experimental verification; especially if the ORF is only a genomic DNA prediction. However, on its own, active transcription is insufficient to prove translation, even with a predicted CDS, and it is established that pseudogenes can exhibit low-level transcription ^15^. While it has inherited the categorisations from UniProt, the neXtprot database has a particular focus on the evidence code system and has set up collaborations to extend experimental support in general ^13^. The outlines of this can be seen in Figure 4.

The categories (expanded on in the neXprot documentation) are as follows:

1. PE1: evidence that includes at least partial Edman sequencing, mass spectrometry (MS) with a threshold of 2 peptides of at least 9 amino-acids, X-ray or NMR structure, protein-protein interaction data or detection by antibodies (Abs).
2. PE2: not proven at protein level but has transcription data (e.g. cDNA, RT-PCR or Northern blots).
3. PE3: probable existence based on orthologues with high similarity scores being found in related species.
4. PE4: no evidence at the protein, transcript, or homology levels.
5. PE5: may be a spurious *in silico* translation of a non-coding transcript.

There is now a community effort to promote more proteins to P1 using both MS and Abs, so we can go into these in more detail. The former has a long history with a proprietary project reporting MS identification of 14,223 human proteins as early as 2004 ^16^. An analogous public effort described the verification of 11,115 Ensembl coding sequences, made available in the first data release of the ProteinAtlas (PA) in 2005 ^17^. By 2017 the Human Proteome Organisation has been extensively engaged in MS initiatives, particularly in regarded to the “missing proteins” (i.e. those still in P2 to P5) that remain refractory to tryptic peptide verification at the necessary stringency. This aspect has been the subject of several recent reviews and so does not need expanding here ^18, 19^.

As another important methodological push, antibody-based proteomics has developed more recently into a large-scale enterprise. This was first described in 2014 as the Human Protein Atlas project with its own associated database ^20^. This has now been extended with the setting up of an International Working Group for Antibody Validation and the accompanying Antibodypedia database ^21^. These have the objective to increase the reproducibility of protein identification and ultimately, as with the MS initiatives, to move more sequences up to the P1 evidence code)

**Figure 5.**
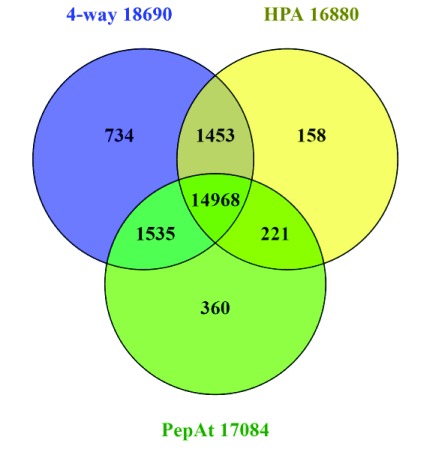
Swiss-Prot cross-reference intersects between the 4-way confirmed set from Figure 3, the Human Protein Atlas (HPA), and the Peptide Atlas.

We can use the categories above to further “slice and dice” cross-referencing to gain more insight into particular subsets (e.g. via downloadable identifier sets for P1to P5). The possible query combinations are many, so we need to frame useful questions. Notably, it is now possible to select proteins supported by PA MS support entries (17,084) or HPA (16,800) or both (15,189) (n.b. numbers differ slightly from those in neXtprot of 18,083 for PA and 16,473 for HPA). In terms of questions, an example that can be posed is “how many proteins, either supported by HPA or PA, overlap with the 4-database consensus set generated in Figure 3?” The result ( Figure 5) effectively intersects the *in silico* with the in *vivo* evidence sets.

As was done for Figure 3, lists from the Venn sections were input to the UniProt ID mapping interface to examine trends. Not all of these can be discussed here, but looking at the unique sets exposed some initially counter-intuitive results. For example, the 4-way only (734) included 214 P1s, but without HPA or PA cross-references. This is because P1 also includes 3D structures and interaction data. Looking at the 152 HPA-only set included 101 at P4 or P5 levels (i.e. unexpectedly high for the implied Ab confirmation which might be expected to push them up to P1). It turns out there is a cross-reference specificity problem from the inclusion of uncertain results. The HPA link (for the 16,800) actually means the protein has been tested (i.e. had an antibody raised against peptide sections) but is not necessarily confirmed. The histochemistry support status, including consistency with two sources of transcript data are commented on in each HPA entry. However, from the HPA download for 16.1, only 10,230 (of the Ensembl proteins as primary identifier) are designated as “approved” or “supported” at the histochemistry level. Examples of evidence complications include the 40-residue of putative protein FAM86JP as the Swiss-Prot entry Q05BU3. Flagged as P5, this shows anomalies including designation as a pseudogene by HGNC (n.b. it has neither GeneID nor an Ensembl cross-reference which excluded it from the 4-way set) and the HPA entry ENSG00000186523-FAM86B1 was flagged as uncertain based on two antibodies. A second example exposes a different problem. The putative uncharacterized protein C7orf76 ( Q6ZVN7) is mapped from UniProt to a different protein in HPA as ENSG00000127922-SHFM1 (i.e. P60896). The miss-mapping appears to be extrinsic to HPA and in this case could be a UniProt < > Ensembl problem (which is why this is not in the 4-way set). It is important to emphasise that none of this is about fault finding, but these examples attest to the technical challenges of evidence classifications and mapping fidelity.

Inspecting the 360 “PepAt” (i.e. PeptideAtlas only) set reveals a different set of interpretive challenges. An example is the smallest of the set at only 11 residues as morphogenetic neuropeptide ( P69208). This has no genomic annotation, but does have an apparent match in PeptideAtlas for the peptide QPPGGSKVILF. The Swiss-Prot entry has its origins in an Edman sequencing result from 1986 and is consequently indicated as “Experimental evidence at protein level”, but has been dropped from neXtprot. A large proportion of the rest of the 360 are immunoglobulin heavy variable and HLA class I histocompatibility antigen chains for which the ability of the PeptideAtlas system to resolve into separate proteins is unclear.

## Small proteins

Back in 2004, it was already mooted that a significant expansion in protein number was likely to occur via the discovery of small ORFs (smORFs). However, this was not supported by Swiss-Prot statistics at that time ^4^. In the intervening decade, the smORF question has surfaced regularly ^22^ and it now overlaps with the two closely related themes of *de novo* protein evolution (i.e. recent non-coding to coding transitions) ^23^ and ribosomal profiling experiments attempting to define the translation of novel smORFs from what was hitherto classified as non-coding RNA ^24^. In addition, the theme of existence evidence discussed above is also relevant, since whatever data support type is being sought (e.g. active transcription plus detection by MS or Abs), the experimental verification of smORFs becomes more difficult.

An obvious approach to this topic is to repeat the exercise first performed in 2004 ^4^, namely splitting the smORF count in Swiss-Prot by create date. By setting a cut-off of 100 residues, the current total is 682/20,168. This can be compared with the corresponding 2009 totals of 612/19,675. This establishes that the proportional smORF content has only risen from 3.1% to 3.3%. In addition, from the latest 2017 size cut, 161 of the 682 do not have an HGNC biotype designation as protein-coding. Many also only have the protein existence support as Edman sequencing reads from the earliest Swiss-Prot releases. These short sequences are difficult to genome map and/or re-confirm by MS, which is why six were recently purged from neXtprot (P.Gaudet personal communication). We are thus presented with a paradox that, despite many reports of putative novel human smORF discovery, very few are crossing the Swiss-Prot evidence threshold for becoming new protein entries.

Notwithstanding, recently confirmed smORF examples have surfaced that are informative from the protein counting viewpoint. The first of these, the apelin receptor early endogenous ligand, was integrated into Swiss-Prot in 2014 (HGNC symbol APLEA, synonyms Elabela, Toddler; see Swiss-Prot P0DMC3 for cross-references including links to the discovery papers). It was in fact “hiding in plain sight” in so far as its full-length cDNA ( AK092578) had been in GenBank since 2008. However, since this sequence translates into eight possible smORFs, the submission process for the high-throughput cloning project (sensibly) chose not to annotate a CDS in the feature lines of this prostate library entry, since there was no basis on which to choose any of the possible translations by protein similarity at that time (although arguably, manual sequence analysis, including TBLASTX, might have given clues). Significantly though, this transcript had originally been annotated in Vega as a Long non-coding RNA (LncRNA) giving rise to speculation that additional cryptic smORFs could be “hiding” in other LncRNAs. Such a second case has in fact been described in 2016 in paper entitled “A peptide encoded by a transcript annotated as long noncoding RNA enhances SERCA activity in muscle”, although the work was done in mouse ^25^. The publication was processed by Swiss-Prot in March 2016 to generate P0DN83 and P0DN84 for a 34 residue mouse and human proteins, respectively.

These two smORFs illustrate a spectrum of evidence differences as follows:

- In terms of transcript support, APLEA has been re-cloned as KJ158076 with a submitted CDS, but this is not yet incorporated in the Swiss-Prot annotation. The DWORF authors mention obtaining cDNAs but have neither deposited human or mouse mRNA accession numbers. There are many TBLASTN matches as supporting evidence for the protein (not withstanding miss-matches, see below) both to mammalian sequences designated as LOC non-coding RNAs and over 30 human expressed sequence tag (EST) mRNAs.
- APLEA has three-way genomic support and a CCDS, while DWORF has no human genome cross reference in Swiss-Prot. The mouse paralogue does have an Ensembl protein mapping ( ENSMUSG00000103476) despite still being flagged as an LncRNA gene in the Mouse Genome Atlas. However, multiple lines of evidence (Southan, unpublished observations) indicate the correct human sequence is the 35 resides represented in ENSG00000240045 (via Vega) as TrEMBL A0A1B0GTW0 (but circularly as this was picked up from Ensembl) and independently as ACT64388 from 2009. The predicted transcript is classified by NCBI as a non-coding LOC100507537.
- Neither APLEA nor DWORF have any cross-references in the seven MS sources in UniProt. Note that APLEA cannot pass the double 9-mer criteria for neXtprot, and DWORF only has a single predicted tryptic peptide. Whether either protein passes the verification threshold for MS datasets in the future remains to be seen.
- Publications for both APELA and DWORF have included Western blots from Abs raised against peptides (but mouse for the latter). However, neither yet has an HPA entry. While the possibility of inclusion in a future update is clear for APELA, there may not only be technical challenges from the small size of DWORF, but also, since HPA uses Ensembl IDs for its primary identifiers, this protein and its transcript would need first to be resolved in a future Ensembl release (n.b. LOC100507537 appears to have somehow parsed HPA transcript data, but this may be a miss-mapping).
- Replication of the basic findings and expanded aspects of *in vivo* function have been consolidated in numerous publications for APELA, including a 2017 paper ^26^. While the experimental characterisation of DWORF rests on one study done with mouse so far ^25^, consolidation of the human protein evidence is to be expected in forthcoming work.

To summarise the implications; the discovery of additional smORFs seems certain, especially given that the putative LncRNA gene count has recently risen to 27,919 ^27^. However, the question remains as to how many will be verified to the evidence level sufficient to enter the major genome and protein portals (even though it will be challenging to obtain Abs and MS verification data). On a continuum of what we might expect between 10, 100 or 1000, the middle estimate seems most likely.

## Pharmacological interaction intersects

**Figure 6.**
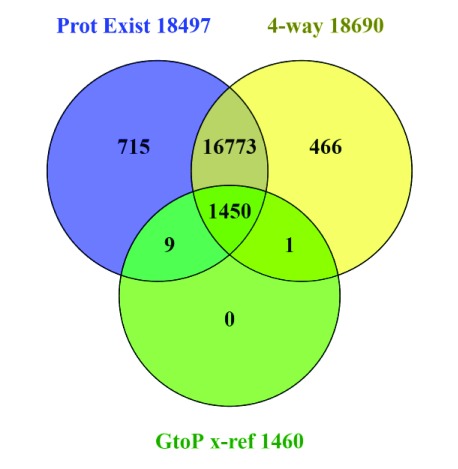
Corroboration of human proteins in Swiss-Prot with ligand interactions in GtoPdb (selected as “GuidetoPHARMACOLOGY” in the Chemistry cross-references). The first of the two intersected lists are labelled as “Prot Exist” with evidence at the transcript and protein levels (i.e. PE1 and PE2 from Figure 4), and the 4-way major source consensus set (i.e. the central panel of Figure 3).

This last section assesses the corroboration of data linkages by existence evidence and other types of concordance. Many of the Swiss-Prot cross-references are related to protein function and other attributes such as tissue distribution or post-translational modification. Others would include pathway membership, protein-protein interactions, Genome Ontology categorisation, disease associations, interactions between enzymes and substrates, drugs and their targets, as well as endogenous ligands for receptor proteins. The advantage of the analyses described above is that results centred on functional categories can be intersected with independent cross-references. This can be exemplified by selecting the curated ligand interactions in the IUPHAR/BPS Guide to PHARMACOLOGY ^28^ (GtoPdb) that are included in the set of five chemistry (interaction) cross-references. The current UniProt has 1,460 human Swiss-Prot records (as defined by the GtoPdb criteria for submitting the links) that have publication-supported molecular interactions. The majority are pharmacologically active small-molecules, but the curated relationships include some protein-protein interactions, for example, antibody ligands directed against cytokine targets (n.b. a proportion of these proteins are derived from a new project as the Guide to Immunopharmacology). The result of the corroboration analysis is shown in Figure 6.

We can see the results of a three way comparison in Figure 6 between existence evidence, four-source convergence and GtoPdb entries. The first feature to note is that not all proteins with existence evidence are in the four-source set, and vice versa. Possible systematic reasons behind this cannot be explored here, but may be related to the points discussed for Figure 5. The key observation for GtoPdb is that, reassuringly, 1,450 entries intersect with both existence evidence and four-source identifiers. Notwithstanding, there are nine intersects with the existence set but not four-source corroborated with one vice versa (i.e. in the four-source set but not evidence-supported). Given that GtoPdb interactions are expert-curated, the result from Figure 6 raises questions about the annotation of the 10 protein entries. These were followed up to establish that the lack of evidence support for P0C264 arises from the absence of an mRNA entry (i.e. it remains a genomic prediction). The existence of this kinase seems well supported (e.g. via CCDS74457), but a cloned cDNA would be an important consolidation. Inspection of the other nine sequences also supported their existence but they all had a mixture of cross-referencing failures that had excluded them from the four-source set. For example, for the aspartyl aminopeptidase, DNPEP ( Q9ULA0) the protein is solidly supported even to the extent of a PDB structure, but the Entrez GeneID is missing (although this is cross-referenced by HGNC). Likewise, the alpha-2B adrenergic receptor, ADRA2B ( P18089) is solidly supported, but in this was missing the Ensembl cross-reference (it turns out from Swiss-Prot update enquiry this was due to an unusual accession number change associated with a TrEMBL to Swiss-Prot transition, Gasteiger, personal communication). In both cases GtoPdb had in fact been manually curated in the correct links for the Entrez Gene ID in Target ID 1559 and Ensembl Gene for Target ID 26, respectively (n.b. the appropriate UniProt corrections have been suggested via the feedback form). This cross-checking for GtoPdb targets thus proved a useful exercise that will be re-visited as our protein content expands.

## Conclusions

Despite over 16 years having elapsed since the first draft human genome, the diversity of current counts indicates that progress towards what the community might consider a gold-standard set of canonical protein sequences, remains frustratingly slow. This is especially so considering that the “zone of equivocality” lies only between an upper bound of ∼ 20,000 and a lower one of ∼18,500. The slow progress towards closure is clearly a reflection of both the inherent biological complexity of protein translation, as well as the challenges of combining automated annotation with various proportions of expert curation needed to define the entire expressed genomic landscape ^29^. There are of course caveats, even with the concept of closure, in so far as recent evidence indicates that each of us, on average have at least 100 protein loss-of function variants (i.e. proteomes are “personal”) ^30^.

The wider bioscience community could be forgiven being puzzled that major global efforts continue to produce different sets of canonical proteins at roughly the same time from the same primary data (leaving aside another layer of yet more inter-source differences in alternative splice and/or initiation forms). Those of us with some insight into the bioinformatic, genomic and proteomic challenges might be more sanguine in our judgment, but the criticism still stands (note also that human is the testbed from which the community needs progress to analogous proteomic closure for at least mouse, rat and Zebrafish). Approaching the question as to why this situation persists and possible solutions, would necessitate a detailed comparison of the underlying assumptions, data processing models and pipeline parameterisations. However, inter-source clustering of explicit protein sequences could make identifying difference more effectively than cross-references alone (e.g. a possible resurrection of the Human Protein Index initiative ^31^).

Regardless of the technical options to solving the problem, substantial resources have been committed over decades by the major gene and protein annotation resources globally. We should thus expect more inter-team collaboration dedicated to harmonising amongst themselves for the mere ∼2000 protein sequences in question (i.e. not many compared to the 0.55 million and 77 million processed in Swiss-Prot and TrEMBL respectively). It could be argued that additional (collective) manual curation would be needed to accomplish this, but the consequent improvement *in silico* concordance could then be consolidated by an expansion of experimental existence verification both *in vitro* and *in vivo*. This could include a supply of expressed protein standards, advances in MS-based proteomics, including sets of synthetic proteotypic peptides for spiking experiments ^32^, deep transcript profiling by RNA-seq and the increased availability of validated antibody reagents.

## Data availability

The data referenced by this article are under copyright with the following copyright statement: Copyright: © 2017 Southan C

Data associated with the article are available under the terms of the Creative Commons Zero "No rights reserved" data waiver (CC0 1.0 Public domain dedication). 

These statistics on protein numbers are presented and compared here in good faith and with implicit expectation that they should be reproducible, including by others who may want to repeat and/or extend these types of analyses. Notwithstanding, this may be confounded by several factors that could give rise to slightly different results (but it is hoped not major discrepancies). The most obvious is data updates that can be as frequently as monthly for some sources (e.g. since the completion of this work UniProt notched up to UniProt release 2017_03 on March 15, 2017 with the human SwissProt count increasing, from Table 1, by 13 proteins to 20,184). Another is the exact form of the queries, which vary between resources, particularly when each selection interface has a different look and feel, different syntactic formats of execution and download lists having different formats of cross-referenced identifier columns. One example is the need to covert UniProt interface queries into the equivalent SPARQL queries in neXtprot as shown below. The UniProt syntax to count HGNC cross-references, as entered in the web query box, is below:

database:(type:hgnc) AND reviewed:yes AND organism:"Homo sapiens (Human) [9606]"

The answer was 19967 (March 2017), but note we need to make to pre-selects for a) species/organism and b) “reviewed” to select Swiss-Prot over TrEMBL. For the neXtProt equivalent cross-reference query, these two pre-selects are not necessary since is human Swiss-Prot derived anyway. The HGNC select has the form below:

select distinct ?entry where {

 ?entry :reference ?ref .

 ?ref :provenance db:HGNC ;

  :accession ?ac.

 filter (regex(?ac,'^HGNC'))

}

In this case the result was 19956. The basic listings from sources used and some of the result sets have been made available as a Figshare data collection ( https://figshare.com/collections/Supplementary_data_for_assessing_the_human_canonical_protein_count/3716413 ^33^). If any reproducibility issues do arise, interested parties are welcome to contact the author. 
