# Supplementary material for: Last rolls of the yoyo: Assessing the human canonical protein count
Source: F1000Res. 2017 Apr 7;6:448. [Version 1] doi: 10.12688/f1000research.11119.1 (PMC5428527; doi:10.12688/f1000research.11119.1)
Supplement: Peer review history

# Peer review: Referee response for version 1

**DOI:** 10.5256/f1000research.11995.r22248

**Published:** 2017-05-09

**Authors:** Sylvain Poux, Lionel Brueza

**Affiliations:** 1 Swiss Institute of Bioinformatics, Lausanne, Switzerland

The article compares the number of canonical proteins encoded by the human genome in different resources, including UniProtKB/Swiss-Prot, HGNC, neXtProt, GeneID, Ensembl or CCDS. The major conclusion is that the number of canonical proteins should be around 19,000 and that, while numbers converge across resources the full canonical human proteome is still not defined.

 This is a good description of the current situation and the article is therefore interesting even if there could be confusion between protein-coding genes and canonical proteins. If the author assumes it is the same, maybe for consistency reason it would be good to mention only protein-coding genes or to explain what the differences are. The author also suggests that an inter-team collaboration could come-up with a finished canonical proteome and seems to ignore the ways the different resources already collaborate. As this has already been raised in the review of E.Bruford, we will not enter into details. The question of the release cycle is also important and should be developed in more details. Many discrepancies are only transitional and only due to the release cycle of the resources compared. As mentioned in the article, neXtProt is built on UniProtKB/Swiss-Prot and differences between these resources are only due to release schedule. But this is also holds true for the other resources and should be emphasized.

 Another issue concerns the methodology of the study. A number of resources compared in this study do not have the same primary mission and it is therefore normal to have discrepancies between them. For example, HGNC is a nomenclature committee and official gene names are assigned when a consensus name is reached in the community. As a consequence, some clear protein-coding genes, such as NSG1 and NSG2 (UniProt P42857 and Q9Y328, respectively) are not yet present in HGNC, because no consensus has been found for these genes. The same is true for CCDS, which aims to provide a consensus sequence for all protein-coding genes: some protein-coding genes are absent from the CCDS set because no consensus has been found for the sequence (for example ELOA3C; UniProt A0A087WX78).

 An alternative approach to assess the number of human protein-coding genes might be to compare portals described in this article with proteomics resources: it might be interesting to investigate the number of peptides that do not match to protein-coding genes in HGNC, UniProtKB/Swiss-Prot or GeneID.

 We think that the article would benefit developing these different points in the discussion.

 There are a number of typos and imprecisions in the text listed below that alter the quality of the manuscript and should be reviewed:

 “are all cross referenced to a single, maximal length, protein entry.”

 It is not absolutely true since the maximal length is one the criteria. However, the relevance of the selected canonical protein in Swiss-Prot in term of expression and biological relevance are also considered among other criteria.

 “the fact that that”

 “the first two are essentially automated pipelines” it is not clear what the author is referring to? Swiss-Prot and HGNC?

 “There is now a community effort to promote more proteins to P1”

 The author uses indifferently PE1 to PE5 and P1 to P5. This could be misleading.

 “indicate the correct human sequence

 is the 35 resides represented in”

 One should read residues instead of resides.

 “The current UniProt has”

 When mentioning the database, prefer UniProtKB

We have read this submission. We believe that we have an appropriate level of expertise to confirm that it is of an acceptable scientific standard, however we have significant reservations, as outlined above.

# Peer review: Referee response for version 1

**DOI:** 10.5256/f1000research.11995.r21692

**Published:** 2017-05-05

**Authors:** Elspeth Bruford

**Affiliations:** 1 European Bioinformatics Institute, European Molecular Biology Laboratory, Hinxton, UK

**General remarks:**

 The author has investigated the question of how many protein coding genes are encoded in the human genome, and come to the conclusion that while protein coding gene counts from a variety of resources do appear to be converging, there are still significant differences. One key aspect the author has maybe not fully appreciated is the considerable level of collaboration already occurring between the cited resources, which can be both advantageous - by reaffirming correct annotations - and disadvantageous - by perpetuating erroneous annotations through multiple resources. At the same time, definitions of biotypes, and membership within in each biotype, do still present differences which groups, including members of the CCDS collaboration, are looking to unify. Furthermore, while different interpretations of available data can of course cause discrepancies (and this is perhaps an area where more work is required by the community to reach agreed standards, for example see PMID 26367542 ^1^), unsurprisingly some resources access different datasets which cause further differences. While collaborations, definitions and data-sharing could be tightened up, there is no doubt that what is most needed is concerted experimental investigation of the remaining putative/hypothetical/dubious protein coding loci that remain within the genome, so that the resulting data can be used to decide upon a definitive biotype for these loci.

 Overall this is discussing an important question but there is a tendency to be rambling in sections and I think the paper needs better organising to highlight some interesting questions the author raises. Some assumptions made about the various projects also need to be corrected, and more attention to detail is required for the numbers quoted to avoid confusing readers.

**Specific remarks:**

 use of "miss-" throughout instead of mis-

 Ensembl, not Ensemble

 Entrez Gene is more widely referred to now as "NCBI Gene" - but variously referenced throughout the ms as "GeneID", "NCBI genome annotation", "RefSeq and Gene", "NCBI Entrez Gene", "GI", "NCBI", "NCBI pipeline automation" etc...

 GENCODE, not GENECODE

 HGNC is HUGO Gene Nomenclature Committee (not Human)

 neXtProt, not neXtprot

**Abstract:**

 I disagree that the only suggestion that total numbers of protein coding genes may rise is from reports of smORFS - as the author discusses later in the paper, even the very few genes reported to date to encode "smORFs" have limited evidence. I would anticipate most increase would come from careful re-annotation using increasing amounts of data (conservation, RNAseq, etc) and from annotation of multiple haplotypes that may cover regions of the human genome that are not currently included in the reference assembly and could be included in the future as alternate loci by the GRC.

**Introduction:**

 While saying that the longest mRNA strategy has data support, it would also be worth mentioning the exception of read-through transcripts which can confuse this strategy significantly.

**Historical Growth:**

 It could also be worth noting that Ensembl and Swiss-Prot/UniProtKb are also coupled as Ensembl sequences that are absent from UniProtKB are imported into UniProtKB/TrEMBL and tagged as part of their human proteome.

 Ensembl's statistics make it very clear the number of proteins encoded by readthrough transcripts and on "alternative sequence", so I don't see how these could be said to "complicate" the figures. The issue of how many of the proteins (and protein coding genes) included on the alt loci are not represented in the primary assembly is however an interesting question.

 Figure 2 shows the Swiss-Prot protein counts divided into total in red and those with protein or transcript evidence in blue - it would be nice to have the 2017 figures actually stated as opposed to having to guesstimate them from the graph.

**Current Counts:**

 The author does not seem to understand the relationship between GENCODE (not GENECODE), Vega/Havana and Ensembl. It is nicely explained on the GENCODE site: https://www.gencodegenes.org/faq.html

 Hence none of these figures are truly independent at all, and any differences between Ensembl and GENCODE figures are likely due to release asynchrony.

 As it is unsurprising that GeneCards, which combines data from a variety of resources, has the largest "protein coding gene count" it is equally unsurprising that the CCDS consortium has the lowest as they are looking for the consensus CDS from Ensembl/Havana (=GENCODE) and RefSeq.

 I disagree with the statement that mapping identifiers across sources can "establish if the protein sequence in pipeline output A is the same as pipeline B", and indeed the author discusses the example of *BACE1 *which shows this is not necessarily true; however, this discrepancy is not due to the mappings themselves or how they are made, but simply due to the methods of protein prediction/selection used in each resource. The mapping may correctly suggest that both pipelines are considering the same genomic locus (in this case the *BACE1 *gene), but agreement on the encoded protein(s) is not guaranteed. This paragraph would be better rephrased to make this clear.

 Typo: if HGNC instantiate then they should also collate (not collates)

**Cross-reference counting:**

 "However, the choice was made here to exemplify just four identifiers, Swiss-Prot accession numbers, HGNC IDs (directly, or via the current gene symbols) Ensembl gene IDs and NCBI Entrez Gene IDs. These were chosen for their global prominence but also methodological complementarity. This derives from the fact that that the first two are essentially automated pipelines (but different), while the second two are primarily manual expert annotation operations (but also different)"

 The first two resources in the list are Swiss-Prot and HGNC, and neither are "essentially automated"; I think the author meant to say "last two" as nowhere else in this paper does he suggest either of these resources rely heavily on automation. As discussed earlier, the Ensembl gene set is a merge of their automated predictions with Havana manual annotations, and the manual annotations make up the vast majority of the protein coding genes. Likewise, the NCBI "Entrez Gene IDs" undergo extensive manual curation, especially for the human set. Therefore I would disagree that *any* of these four resources are "essentially automated pipelines".

 Figure 3 is very confusing with all of the "zero" segments - this figure would make more sense if it was an "all by all" comparison, as opposed to being based solely on the Swiss-Prot dataset. In fact I would venture that a simple table would be more readable, and as stated later in the paper this is a "Venn-type set(s) that generally end(s) up being more confusing than illuminating." Further, the numbers listed in Fig 3 do not correspond with those in the text - in Table 1 20,617 mappings were listed for "GeneID"/NCBI, whereas here there are 18,896, a difference of 1,721, not 2,923 as stated in the text. And for HGNC 19,957 is 924 higher than the 19,033 listed in Table 1, not 905 higher as stated.

 The reason for the increase in mappings to HGNC IDs is more likely the inclusion of mappings to loci that HGNC do not regard as protein-coding, such as immunoglobulin light chain segments, than it is due to Swiss-Prot having more than one HGNC ID in any given record. In fact this explanation is discussed in the next paragraph where the types of loci enriched in specific segments are discussed, such as immunoglobulin light chain segments and endogenous retroviruses (note again the NCBI pipeline is referred to as "automation" which I do not think is a fair representation). I think it would make more sense for these two paragraphs to be rewritten to present the reasons more coherently.   Perhaps it also would have made a better comparison to limit the Swiss-Prot data to loci that ALL four resources regard as protein coding, or to at least present how many of the loci in some segments of the diagram each resource individually considers as protein coding? This would also have made a comparison with the figures in Table 1 more valid, as currently the figures in Table 1 represent a different set to those being compared in Figure 3. Finally, the zero figures in the Ensembl (not Ensemble!)/Swiss-Prot and NCBI/Swiss-Prot segments must reflect their efforts to map between resources, though I am surprised there are no differences at all, even due to update cycle asynchrony? These figures certainly do not result from HGNC importing everything that NCBI and Ensembl annotate automatically (which I note Michael has suggested in his review), as this is definitely not the case. In the next paragraph 19,035 rows are quoted (twice) for HGNC data, but again this does not tally with the figure of 19,033 quoted in Table 1 for HGNC protein coding loci.

**Existence Evidence:**

 In the figures quoted with evidence from Peptide Atlas for Swiss-Prot and neXtProt (17,084 vs 18,083) I would disagree that this could be described as a "slight" difference; this is nearly 1000 loci, which is at least 5% of the protein coding loci in the genome, even using the highest of the counts cited in this paper. What is the reason for this difference, it would be interesting to know. In the next paragraph the figure of 152 is quoted for the HPA-only set, but from Figure 5 this looks to be 158. Which is correct?

 Typo - "...complications include the 40-residue **of** putative protein FAM86JP...".

 Also note that while FAM68JP does not have cross-reference to NCBI Gene or Ensembl from Swiss-Prot these can be found in HGNC and NCBI Gene. The last paragraph of this section again mentions the issue of IG chains, which most resources do not class as "protein coding".

**Small Proteins:**

 HGNC symbol is APELA (not APLEA).

 Typo: "...to generate P0DN83 and P)DN84 for **a** 34 residue mouse and human proteins..."

 The name of the second smORF (DWORF) is not actually mentioned until it is listed in the bullet points, it would be good to introduce "DWORF" by name in the paragraph above. I do not agree with the author that from the (paucity of) evidence cited for these examples that it is therefore "certain" that additional smORfs will be discovered, I think "likely" would be more appropriate. 

**Data Availability:**

 When discussing data update cycles, note that HGNC have daily updates and I think the same can be said of NCBI Gene, so these are both far more frequently than monthly.

 Again the figure quoted in the text does not match numbers given in Figure 3: this section says that the query for HGNC cross-references  gave 19967, while Fig. 3 quotes 19957.

 Typo: "...pre-selects are not necessary since **is** human Swiss-Prot derived..."

I have read this submission. I believe that I have an appropriate level of expertise to confirm that it is of an acceptable scientific standard, however I have significant reservations, as outlined above.

## References

[1] BrufordEALaneLHarrowJ: Devising a Consensus Framework for Validation of Novel Human Coding Loci. *J Proteome Res*.2015;14(12) : 10.1021/acs.jproteome.5b00688 4945-8 10.1021/acs.jproteome.5b00688 26367542PMC4765950

# Peer review: Referee response for version 1

**DOI:** 10.5256/f1000research.11995.r21691

**Published:** 2017-04-18

**Authors:** Michael Tress

**Affiliations:** 1 Structural Biology and Bioinformatics Programme, Spanish National Cancer Research Centre (CNIO), Madrid, Spain

**Abstract:**

 “In 2004, when the protein estimate from the finished human genome was only 24,000, the surprise was compounded as reviewed estimates fell to 19,000 by 2014. “

 This makes no sense; it seems to be missing a large chunk.

 “miss-annotation“

**Introduction:**

 “This has its origins in the Swiss-Prot approach to protein annotation whereby protein sequence differences arising from the same genomic locus either by alternative splicing or alternative initiations (or permutations of both) and/or genetic variants, are all cross referenced to a single, maximal length, protein entry ^8^.“

 This is not strictly true, SwissProt does NOT divide up all proteins from the same gene in different entries (TMPO for example). Here you have to be clear that SwissProt does this most of the time.

 “Importantly, while this was originally introduced as the curatorial strategy of choosing the longest mRNA for an entry, it actually turns out to have post- genomic data support, not only in the form that coding-loci express a single main protein (i.e. that most predicted alternative transcripts may not be translated), but also that in most cases this is the max-exon form (i.e. the curatorial choice actually seems to be the biological “default”) ^9^.”

 Strictly speaking this is true, the longest SwissProt form is the biological default in most cases. But it is purely technical and is not the best way of selecting the biological default. The way this paragraph is written makes it sound like it is. Better to say:

 “not only in the form that coding-loci express a single main protein [ref to Ezkurdia et al, JPR] (i.e. that most predicted alternative transcripts may not be translated), but also that in most cases this max-exon form (i.e. the curatorial choice) actually coincides with the biological “default”) ^9^.”

 Ref: Ezkurdia I, Rodriguez JM, Carrillo-de Santa Pau E, Vázquez J, Valencia A, Tress ML. Most highly expressed protein-coding genes have a single dominant isoform. J Proteome Res. 2015 Apr 3;14(4):1880-7. doi: 10.1021/pr501286b. ^1^

**Historical Growth:**

 “One of these is the inclusion of “alternative sequence”, referring to genomic sections that differ from the primary contiguous assembly. The current release of Ensembl (87.38) species 2,541 proteins in this category, but it is not clear which of these are just variants of those derived from the primary assembly.”

 Alternative sequence genes are not included in the Ensembl reference counts.

 principle = principal

 GENCODE not GENECODE!

 And GENCODE, VEGA and Ensembl ARE merged and have been for a number of years.

 VEGA is annotated by the HAVANA group (part of the GENCODE Consortium), not Havanna. 

 It’s also worth pointing out that Ensembl (since it is now merged with GENCODE) is essentially a manually curated annotation too with manual curations coming from the HAVANA team.

 “Consensus Coding Sequence (CCDS) project. These correspond to a core set of proteins annotated as having full length transcripts that exactly match reference genome coordinates.”

 In fact CCDS transcript models need to exactly match between RefSeq and Ensembl/GENCODE, which explains why CCDS is the smallest set. This is actually an important caveat for the next paragraph, as might be imagined.

**Cross-reference counting:**

 It is also worth mentioning that these are the only four independent sets, in that Vega and GENCODE merge into Ensembl, NextProt is UniProt and GeneCards and CCDS are essentially intersections and unions of different subsets.

 Ensembl, not Ensemble.

 “The explanation is that Ensemble and NCBI Gene have consolidated mapping reciprocity for proteins in Swiss-Prot (but, as mentioned above, many proteins from these two sources are still nominally “outside” Swiss-Prot).”

 I think what it really says is that genes annotated in both Ensembl and SwissProt are automatically included in HGNC.

 “GENCODE and Vega do not currently have cross-references inside Swiss-Prot”

 Because GENCODE/VEGA == Ensembl

 “forth” - fourth

**Existence evidence:**

 “However, on its own, active transcription is insuficient to prove translation, even with a predicted CDS”

 Maybe given the proliferation of such papers it might be worth pointing out that neither is ribosome profiling evidence …

 “in regarded to” in regard to

 “As was done for Figure 3, “

 I think this whole paragraph could be written more carefully. I can follow it, but I suspect most people wouldn't. The data sets being compared need to be introduced specifically (again) and the numbers cross-checked. The examples are interesting, but:

 “A second example exposes a different problem. The putative uncharacterized protein C7orf76 (Q6ZVN7) is mapped from UniProt to a different protein in HPA as ENSG00000127922- SHFM1 (i.e. P60896). The miss-mapping appears to be extrinsic to HPA and in this case could be a UniProt < > Ensembl problem (which is why this is not in the 4-way set).”

 Actually the problem stems from the fact that Ensembl annotates a single gene  (now called SEM1) for these coordinates, while RefSeq has two (those listed in the paper). I have looked at this case before and wrote “RefSeq has two genes for SHFM1; RefSeq is right”. I am not 100% sure that it is, but if it is one gene, it looks to be a gene that has two ORFs and hence it makes sense that UniProt has two entries.

 Small proteins

 “APLEA” = APELA

 Also worth pointing out the conservation all the way back to Danio for APELA. In fact cross-species conservation studies currently being undertaken by Ensembl may unearth some “missing” smORFs.

 “not withstanding miss-matches”

 “miss-mapping”

 “However, inter-source clustering of explicit protein sequences could make identifying difference more effectively than cross- references alone (e.g. a possible resurrection of the Human Protein Index initiative ^31^).”

 Nooooo, do not resurrect the IPI, it died for good reasons. I don’t believe that we need any more competing (and only superficially communicating) bodies in the field.

 “It could be argued that additional (collective) manual curation would be needed to accomplish this”

 This is nice in principal, but manual curation is VERY subjective. For many genes whether it is annotated as coding or not is based on the balance of probabilities and each annotator has his/her own balance of probabilities. What is needed is more and better information for the corner cases.

**General Comment:**

 There are a lot of distinct sets being compared in the figures. I would name and define the sets clearly in the text when possible, otherwise readers will struggle to see what is being compared.

I have read this submission. I believe that I have an appropriate level of expertise to confirm that it is of an acceptable scientific standard.

## References

[1] EzkurdiaIRodriguezJMCarrillo-de Santa PauEVázquezJValenciaATressML: Most highly expressed protein-coding genes have a single dominant isoform. *J Proteome Res*.2015;14(4) : 10.1021/pr501286b 1880-7 10.1021/pr501286b 25732134PMC4768900
